# Gender Differences in Frontotemporal Dementia: Clinical, Brain Structural, White Matter Hyperintensity, and Brain Metabolic Features

**DOI:** 10.1002/alz.090287

**Published:** 2025-01-09

**Authors:** Min Chu, Li Liu, Binbin Nie, Liyong Wu

**Affiliations:** ^1^ Xuanwu Hospital, Capital Medical University, Beijing China; ^2^ Beijing Engineering Research Center of Radiographic Techniques and Equipment, Institute of High Energy Physics, Chinese Academy of Sciences, Beijing China

## Abstract

**Background:**

Gender is a factor influencing the clinical and radiological heterogeneity in dementia. However, gender differences in frontotemporal dementia (FTD) are still underexplored. This study aims to investigate the gender heterogeneity in FTD patients and its association with clinical and imaging features.

**Method:**

FTD patients from two independent cohorts were included in this study, one from our center and the other from the Frontotemporal Lobar Degeneration Neuroimaging Initiative (FTLDNI). For clinical characteristics analysis, our center enrolled 64 male and 70 female FTD patients, while FTLDNI selected 91 male and 69 female patients. For brain structural analysis, both our center and FTLDNI included 40 males and 49 females. In the white matter hyperintensity analysis, our center enrolled 21 male patients and 26 female patients, while FTLDNI included 76 male and 61 female patients. In metabolic analysis, only 21 male and 31 female patients were included in our center.

**Result:**

In the Chinese FTD cohort, females had a shorter education year than males, while males had more severe executive dysfunction and hallucinations and agitation symptoms. Males had a higher proportion in the behavioral subtype, while females higher in the language subtype in FTLDNI. Female patients had more significant gray matter atrophy, particularly in the frontal and cingulate regions, compared to male patients. As for white matter hyperintensities, there were no significant differences in quantity and volume between the two genders. Regarding gray matter metabolism, despite females showing more severe gray matter atrophy, they seemed to have better brain reserve function. The atrophy of the left frontal and basal ganglia in females is significantly correlated with cognitive decline, while males exhibit a link between anterior cingulate atrophy and executive dysfunction. Although a significant mediating model was not found, gender has a tendency mediating effect on visual‐vocabulary testing through the fronto‐temporal‐parietal brain region.

**Conclusion:**

This study describes the clinical and imaging differences between genders in FTD, which might be attributed to gender influencing different neural circuits. Gender differences need to be considered in precision therapy and clinical trial design.

